# Lymphocytic Vasculopathy With Cytoplasmic Antineutrophil Cytoplasmic Antibody Positivity After Upper Respiratory Infection: A Case Report

**DOI:** 10.7759/cureus.102428

**Published:** 2026-01-27

**Authors:** Caden L Jones, Cameron Sandefur

**Affiliations:** 1 Internal Medicine, Edward Via College of Osteopathic Medicine, Monroe, USA; 2 Internal Medicine, Willis Knighton Pierremont Hospitalist Group, Shreveport, USA

**Keywords:** cutaneous vasculitis, lymphocytic vasculopathy, petechiae, positive c-anca, purpura

## Abstract

Cutaneous manifestations of vasculitic or vasculopathic processes can pose a diagnostic challenge, particularly when systemic symptoms are absent. We report a case of an adult patient who presented with nonpainful petechiae and blue-purple discoloration of the lower extremities three days after a self-limited upper respiratory infection. Laboratory testing revealed elevated erythrocyte sedimentation rate, white blood cell count, and C-reactive protein, as well as positive cytoplasmic antineutrophil cytoplasmic antibody (c-ANCA) titers. No other systemic findings or organ involvement were identified. A punch biopsy of the affected skin demonstrated vascular changes with a differential diagnosis that included lymphocytic vasculopathy. The patient was managed with short-course systemic corticosteroids and close observation as the lesions gradually resolved without complications. This case highlights an atypical, skin-limited c-ANCA-associated lymphocytic vasculopathy, emphasizing the importance for physicians to maintain vigilance for subtle cutaneous signs of autoimmune or inflammatory disease even in the absence of systemic involvement.

## Introduction

Although cutaneous manifestations of vasculitic and vasculopathic disorders can be difficult to diagnose due to numerous conditions with similar presentations [1], the presence of bilateral petechial and purpuric lesions on the lower extremities can provide an important clinical clue suggestive of an underlying vasculitic process [2]. While leukocytoclastic vasculitis remains the most common small-vessel inflammatory process involving the skin, lymphocytic vasculopathy is an uncommon and often underrecognized entity characterized by lymphocytic reaction with vessel occlusion rather than neutrophilic infiltration of vessel walls [3]. Differentiating these two processes is critical, as their clinical implications, etiologies, and management strategies differ.

Cutaneous vasculitis refers to inflammation of the small- to medium-sized blood vessels within the skin, resulting in vessel wall damage and subsequent leakage of red blood cells into the surrounding tissue [1]. Cutaneous vasculitis commonly manifests as palpable purpura, petechiae, or ulcerative lesions whose distribution and severity correspond to the depth and caliber of the affected vessels [2]. The condition represents a broad spectrum of underlying etiologies, ranging from primary idiopathic processes to secondary causes such as infection, medication exposure, or systemic autoimmune disease [4].

The current literature describes lymphocytic vasculopathy as a histopathologic finding that may accompany autoimmune, infectious, neoplastic, or drug-induced conditions, yet it can also occur as an idiopathic, self-limited process [3]. The term vasculopathy encompasses noninflammatory vascular injury resulting from occlusive phenomena such as thrombosis, embolism, cryoprotein deposition, increased blood viscosity, or proliferative changes within the vessel wall [5]. Among these, livedoid vasculopathy represents a more frequently encountered immune-mediated form, characterized by chronic, relapsing, thromboembolic occlusion of the dermal microvasculature of the lower extremities [5]. In contrast, the lymphocytic vasculopathy suggested by this case reflects a rare, predominantly lymphocyte-driven vascular process with a self-limited and less clearly defined clinical course.

Previous studies have focused largely on systemic antineutrophil cytoplasmic antibody (ANCA)-associated vasculitides, where multiorgan involvement and significant morbidity are common [6]. Although ANCA positivity is classically associated with small-vessel vasculitides, it has also been documented across a wide spectrum of other conditions [7]. ANCA reactivity may occur in autoimmune diseases such as systemic lupus erythematosus and rheumatoid arthritis, in chronic infections and endocarditis, in inflammatory bowel disease, and in certain hematopoietic malignancies [7]. Additionally, ANCA development has been reported as an adverse effect of various pharmacologic agents and, more recently, in association with COVID-19 infection [7]. However, the presence of cytoplasmic antineutrophil cytoplasmic antibody (c-ANCA) in a patient exhibiting isolated cutaneous lymphocytic vasculopathy without systemic features remains uncommonly reported, underscoring the diagnostic challenge and clinical relevance of this case. Consequently, limited data exist to guide clinicians in evaluating or managing patients who exhibit positive autoimmune serologies but exclusively cutaneous disease.

This case contributes to the existing literature by describing an adult male who developed bilateral, nonpainful, violaceous discoloration of the lower extremities following a mild upper respiratory illness, in whom laboratory and imaging studies ruled out systemic vasculitis or infection. The subsequent biopsy suggested a lymphocytic vasculopathy rather than true vasculitis, and the patient demonstrated improvement following corticosteroid therapy. This report underscores an important diagnostic consideration in patients presenting with cutaneous purpura and elevated inflammatory markers that a positive c-ANCA does not always signify systemic vasculitis. By highlighting a skin-limited, reversible presentation consistent with lymphocytic vasculopathy, this case expands awareness of an atypical yet clinically relevant differential diagnosis for osteopathic and allopathic clinicians alike.

## Case presentation

A 33-year-old White male with cerebral palsy and associated bilateral lower extremity deformities, who primarily uses a wheelchair but is able to ambulate with crutches, presented with a three-day history of nonpainful, non-palpable petechial and purpuric discoloration involving both feet, more pronounced on the left. The lesions appeared three days after resolution of an unspecified mild upper respiratory infection, which did not increase sedentary behavior beyond his baseline. The patient denied fever, chills, arthralgias, dyspnea, abdominal pain, or other systemic symptoms. There were no recent medication changes, vaccinations, or exposures, and he denied tobacco, alcohol, or illicit drug use.

On initial examination, the patient was afebrile and hemodynamically stable. Bilateral 2+ pitting edema was noted distal to the lower third of the tibial shafts, with non-blanching petechial and violaceous patches most prominent on the dorsum of both feet, left greater than right. Distal pulses (dorsalis pedis) were 2+ bilaterally, and capillary refill was normal. No ulceration, nodules, or necrosis were initially observed. The remainder of the physical examination was unremarkable.

Initial laboratory evaluation demonstrated elevated inflammatory markers, including an erythrocyte sedimentation rate (ESR) of 41 mm/hr (reference range = 0-20 mm/hr) and a C-reactive protein (CRP) level of 110.7 mg/L (reference range < 0.5 mg/L), both markedly above reference range. White blood cell count was elevated at 13.1 thousand/uL (reference range = 3.1-9.7 thousand/uL), while renal function (blood urea nitrogen, creatinine, estimated glomerular filtration rate), liver enzymes, urinalysis (including urine specific gravity, protein, white and red blood cells, glucose, and casts), coagulation profile, albumin, and electrolytes were within normal limits. Urine and blood cultures drawn on hospital day one yielded no growth. Indirect immunofluorescence assay showed c-ANCA titers of 1:8 (reference range: negative) with anti-Proteinase 3 antibody (PR3) using enzyme-linked immunosorbent assay (ELISA) of 0.8 (reference range <0.4), while perinuclear ANCA (p-ANCA), antinuclear antibody (ANA), rheumatoid factor, and cryoglobulins were negative. Infectious testing, including HIV, hepatitis A/B/C, SARS-CoV-2, influenza A/B, respiratory syncytial virus, and group A streptococcus, was negative.

Imaging studies, summarized in Table 1, included a computed tomographic angiogram (CTA) of the abdomen and pelvis, which demonstrated no vascular abnormalities, and bilateral lower extremity duplex ultrasonography showing patent vessels and no evidence of thrombosis or occlusion. Chest radiography was unremarkable.

**Table 1:** Imaging studies revealed no evidence of vasculitis, occlusion, or thrombosis, supporting a localized rather than systemic vascular process. CT = computed tomography; PA = posteroanterior.

Imaging modality	Anatomic region	Findings	Interpretation
CT angiogram (abdomen and pelvis)	Abdominal vasculature	No vascular occlusion, aneurysm, or inflammatory changes identified	Normal; excludes mesenteric or large-vessel vasculitis
Lower extremity duplex ultrasound	Bilateral lower extremities	No evidence of arterial or venous thrombosis; biphasic flow maintained throughout	Normal; excludes deep venous thrombosis or arterial occlusion
Chest radiograph (PA and lateral)	Lungs, mediastinum, heart	Clear lung fields; no infiltrates, effusions, or cardiomegaly	Normal; excludes pulmonary involvement of systemic vasculitis

**Figure 1:**
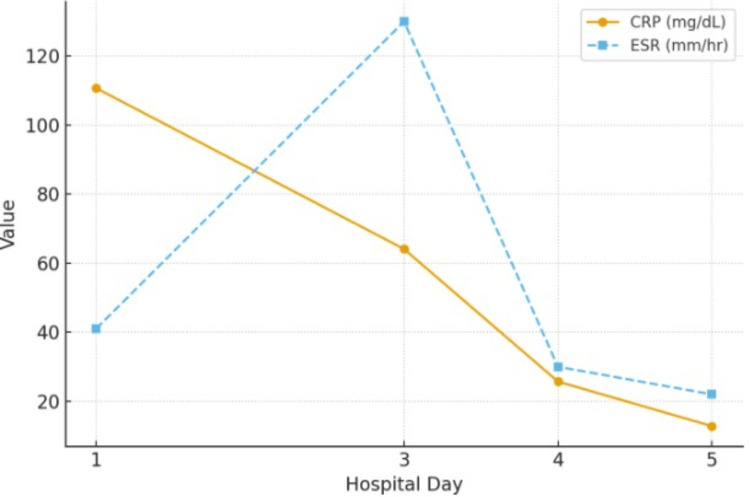
Trend of inflammatory markers (CRP and ESR) over the hospital course. Both markers were markedly elevated on admission and decreased progressively with clinical improvement, consistent with resolving inflammation. CRP = C-reactive protein; ESR = erythrocyte sedimentation rate. Reference ranges: CRP < 0.5 mg/dL; ESR 0–20 mm/hr.

Empiric intravenous vancomycin and cefepime were initiated and continued until the infectious etiology was ruled out on the third day of admission. Deep venous thrombosis (DVT) prophylaxis with subcutaneous enoxaparin 40 mg once daily was also started on admission. On hospital day two, three days from the onset of lesions, general surgery performed a 4 mm punch biopsy on the dorsal left foot lesion. By hospital day three, given persistently elevated inflammatory markers, prednisone 40 mg twice daily was started. By hospital day four, visible improvement in the cutaneous lesions and swelling was observed, with corresponding declines in ESR and CRP levels following corticosteroid initiation (Figure 1).

Histopathologic examination of the biopsy specimen revealed lobular proliferation of vessels within the papillary dermis with intravascular thrombi, hemosiderin deposition, and extravasated erythrocytes without neutrophilic infiltration. Periodic acid-Schiff (PAS) stains were negative for fungal elements, and iron stains confirmed dermal hemosiderin deposition. Taken in conjunction with the clinical presentation, these histopathologic findings were consistent with primary lymphocytic vasculopathy; however, the absence of direct immunofluorescence staining limited definitive diagnostic confirmation.

**Figure 2:**
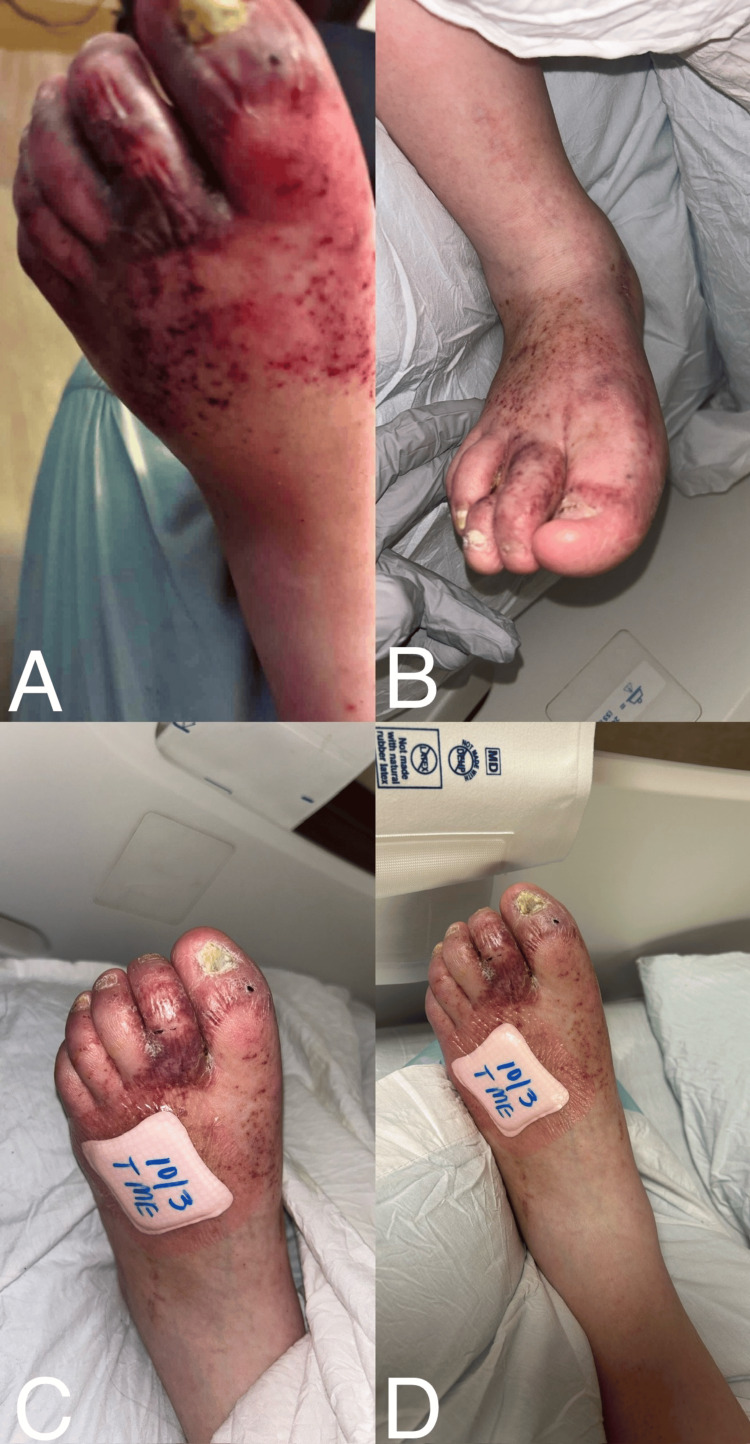
Serial photographs demonstrating the initial presentation and subsequent regression of violaceous and petechial lesions. (A) Initial presentation showing diffuse non-blanching petechiae and violaceous discoloration over the dorsal left foot and toes. (B) Right foot prior to initiation of steroid therapy, with progression of erythema. (C, D) Gradual improvement following corticosteroid treatment, with decreasing erythema and fading of petechiae.

The patient’s lesions continued to improve alongside corticosteroid therapy (Figures 2C, 2D), and near-resolution of discoloration was observed at follow-up compared with the baseline appearance at presentation, characterized by non-palpable, violaceous, purpuric, and petechial patches with associated edema over the dorsum of both feet (Figures 2A, 2B). However, the temporal association between therapy and clinical improvement does not establish causality, and improvement may reflect spontaneous resolution. No systemic manifestations developed during or after treatment. The patient was discharged home on a tapering course of oral prednisone and scheduled for outpatient rheumatology follow-up for continued evaluation and management.

The patient provided written informed consent on hospital day two, using paper consent forms. Consent was obtained by Caden Jones, an author of this case report. The patient granted permission for the use of all clinical information relevant to the case, as well as written permission for the publication of all accompanying clinical photographs. All consent materials were completed in accordance with institutional and journal guidelines for the publication of information and images.

## Discussion

This case highlights the diagnostic complexity of differentiating cutaneous vasculitis from lymphocytic vasculopathy, particularly in patients who present with localized skin findings and positive autoimmune serologies. The presence of elevated inflammatory markers in this setting can further obscure the diagnosis and prompt an extensive, and often unnecessary, systemic workup. While ANCA positivity, especially c-ANCA, is classically associated with systemic small-vessel vasculitides such as granulomatosis with polyangiitis (GPA), a positive result alone is insufficient for diagnosis and requires histologic confirmation [8].

Histopathologic evaluation remains the cornerstone of distinguishing true vasculitis from vasculopathy. Leukocytoclastic vasculitis demonstrates fibrinoid necrosis, vessel wall disruption, and neutrophilic infiltration with leukocytoclasia, whereas lymphocytic vasculopathy is characterized by vessel wall involvement predominantly by lymphocytes, associated hemosiderin deposition, and intravascular thrombi in the absence of neutrophilic debris. Because the resolution phase of acute leukocytoclastic vasculitis is characterized by decreased neutrophils and increased mononuclear cells and can closely mimic the histologic appearance of lymphocytic vasculopathy, a precise correlation with the clinical timeline is essential when considering a diagnosis [9]. In this patient, biopsy findings of dermal vascular proliferation, intervascular thrombi, and hemosiderin deposition without neutrophilic infiltrate with an acute onset of cutaneous findings favored a lymphocytic vasculopathy, a process thought to represent a low-grade, possibly immune-mediated, vascular reaction rather than a destructive vasculitis.

The pathogenesis of lymphocytic vasculopathy remains incompletely understood. Proposed mechanisms include immune complex deposition [10], endothelial injury secondary to infection [10], and localized vascular stasis or pressure changes, particularly in dependent extremities [11]. The patient’s history of chronic lower extremity deformities and improvement with elevation suggests that venous stasis may have contributed to vascular compromise and cutaneous hypoxia, creating a milieu for secondary immune activation. The transient nature of the lesions, the absence of systemic findings, and the rapid response to corticosteroid therapy support a reactive, self-limited process rather than a primary systemic vasculitic disorder.

Previous studies have documented ANCA positivity in a subset of patients without systemic vasculitis, sometimes triggered by infections or inflammatory conditions [8]. In such cases, ANCA titers may reflect transient immune activation rather than pathogenic autoimmunity, underscoring the importance of interpreting serologic results in the appropriate clinical context. Nevertheless, ANCA positivity remains clinically meaningful, as it shows established correlations with vasculitic disease such as GPA, demonstrating up to 73% sensitivity and 99% specificity [12].

Therapeutically, corticosteroids remain the mainstay for vasculopathies when biopsy and clinical features suggest immune-mediated pathology [1]. Empiric antibiotic therapy, as initiated in this case, is common when infection cannot be excluded early; however, once cultures and imaging are negative, de-escalation of antibiotics is warranted [13].

From an educational standpoint, this case emphasizes the importance of clinicopathologic correlation when evaluating purpuric or petechial skin lesions. Reliance on serologic markers alone can lead to diagnostic misclassification and overtreatment. Recognition of lymphocytic vasculopathy as a potential mimic of vasculitis allows for more judicious use of systemic immunosuppressive therapy.

## Conclusions

This case highlights an uncommon presentation consistent with isolated c-ANCA-positive lymphocytic vasculopathy manifesting as self-limited purpura without systemic involvement. The findings emphasize the value of correlating clinical, laboratory, and histopathologic data when evaluating suspected vasculitic processes. Recognition of this entity may support more accurate diagnostic reasoning and patient-centered management within osteopathic and allopathic medical practice. Awareness of cutaneous-limited c-ANCA-associated vasculopathic processes may help clinicians avoid unnecessary invasive testing or extensive immunosuppressive therapy while ensuring appropriate monitoring for potential systemic disease.
